# Investigating Older Adults’ Perceptions of AI Tools for Medication Decisions: Vignette-Based Experimental Survey

**DOI:** 10.2196/60794

**Published:** 2024-12-16

**Authors:** Sarah E Vordenberg, Julianna Nichols, Vincent D Marshall, Kristie Rebecca Weir, Michael P Dorsch

**Affiliations:** 1 College of Pharmacy University of Michigan Ann Arbor, MI United States; 2 Sydney School of Public Health Faculty of Medicine and Health University of Sydney Sydney Australia; 3 Institute of Primary Health Care (BIHAM) University of Bern Bern Switzerland

**Keywords:** older adults, survey, decisions, artificial intelligence, vignette, drug, pharmacology, pharmaceutic, medication, decision-making, geriatric, aging, surveys, attitude, perception, perspective, recommendation, electronic heath record

## Abstract

**Background:**

Given the public release of large language models, research is needed to explore whether older adults would be receptive to personalized medication advice given by artificial intelligence (AI) tools.

**Objective:**

This study aims to identify predictors of the likelihood of older adults stopping a medication and the influence of the source of the information.

**Methods:**

We conducted a web-based experimental survey in which US participants aged ≥65 years were asked to report their likelihood of stopping a medication based on the source of information using a 6-point Likert scale (scale anchors: 1=not at all likely; 6=extremely likely). In total, 3 medications were presented in a randomized order: aspirin (risk of bleeding), ranitidine (cancer-causing chemical), or simvastatin (lack of benefit with age). In total, 5 sources of information were presented: primary care provider (PCP), pharmacist, AI that connects with the electronic health record (EHR) and provides advice to the PCP (“EHR-PCP”), AI with EHR access that directly provides advice (“EHR-Direct”), and AI that asks questions to provide advice (“Questions-Direct”) directly. We calculated descriptive statistics to identify participants who were extremely likely (score 6) to stop the medication and used logistic regression to identify demographic predictors of being likely (scores 4-6) as opposed to unlikely (scores 1-3) to stop a medication.

**Results:**

Older adults (n=1245) reported being extremely likely to stop a medication based on a PCP’s recommendation (n=748, 60.1% [aspirin] to n=858, 68.9% [ranitidine]) compared to a pharmacist (n=227, 18.2% [simvastatin] to n=361, 29% [ranitidine]). They were infrequently extremely likely to stop a medication when recommended by AI (EHR-PCP: n=182, 14.6% [aspirin] to n=289, 23.2% [ranitidine]; EHR-Direct: n=118, 9.5% [simvastatin] to n=212, 17% [ranitidine]; Questions-Direct: n=121, 9.7% [aspirin] to n=204, 16.4% [ranitidine]). In adjusted analyses, characteristics that increased the likelihood of following an AI recommendation included being Black or African American as compared to White (Questions-Direct: odds ratio [OR] 1.28, 95% CI 1.06-1.54 to EHR-PCP: OR 1.42, 95% CI 1.17-1.73), having higher self-reported health (EHR-PCP: OR 1.09, 95% CI 1.01-1.18 to EHR-Direct: OR 1.13 95%, CI 1.05-1.23), having higher confidence in using an EHR (Questions-Direct: OR 1.36, 95% CI 1.16-1.58 to EHR-PCP: OR 1.55, 95% CI 1.33-1.80), and having higher confidence using apps (EHR-Direct: OR 1.38, 95% CI 1.18-1.62 to EHR-PCP: OR 1.49, 95% CI 1.27-1.74). Older adults with higher health literacy were less likely to stop a medication when recommended by AI (EHR-PCP: OR 0.81, 95% CI 0.75-0.88 to EHR-Direct: OR 0.85, 95% CI 0.78-0.92).

**Conclusions:**

Older adults have reservations about following an AI recommendation to stop a medication. However, individuals who are Black or African American, have higher self-reported health, or have higher confidence in using an EHR or apps may be receptive to AI-based medication recommendations.

## Introduction

Approximately 90% of adults 65 years and older (“older adults”) in the United States take at least 1 prescription medication, two-thirds take at least 1 dietary supplement, and one-third take at least 1 over-the-counter (OTC) medication regularly [1,2]. Medications are an important tool to prevent and treat diseases. However, up to one-half of older adults take at least 1 inappropriate medication, which can lead to drug-drug interactions, adverse events, additional medical appointments, emergency department visits, functional decline, reduced quality of life, and increased health care costs [3].

Older adults frequently report that they prefer to participate in shared decision-making [4]. Therefore, they may discuss prescription or OTC medications with physicians or pharmacists as they trust the information provided [5,6]. While these conversations allow patients to receive personalized recommendations, many health care professionals are experiencing high workload and burnout, which interferes with their ability to engage in patient-centered conversations about medications [7,8]. Older adults are increasingly using technology such as smartphone apps and telehealth [9]. Therefore, technology may be an alternative or complementary strategy for older adults to receive personalized medication advice, particularly given the limited time that health care professionals have available with each patient [10].

SEV and MPD sought to address the gap in technological tools for patients related to OTC medications by creating an electronic decision algorithm prototype to help adults identify safe and effective treatments for common ailments [11]. The tool required that participants answer questions about themselves and their symptoms and were given an OTC product recommendation or encouraged to seek advice from a health care professional. Given the complexity and time-intensiveness of manually building and maintaining a comprehensive database, it did not contain detailed information about individual medications (eg, dosage forms, directions for use, and side effects). When the prototype was shared with adults during semistructured interviews, participants reported that they would likely use the technology. However, they simultaneously raised concerns about the accuracy and comprehensiveness of the information, data storage, and accessibility while aligning with broader social and ethical concerns about using technology [12].

While adults reported being interested in using technology to support medication decisions, SEV and MPD quickly identified that creating and maintaining a knowledge base for more than 300,000 OTC medications in the United States would not be feasible [13]. However, the public release of a large language model (LLM) in 2022 revealed how artificial intelligence (AI) can revolutionize how diseases are diagnosed, develop patient-centered treatment plans, and increase patient engagement [14,15]. For example, it is able to provide high-quality, empathetic responses to patient questions [16]. It can also readily provide clarifying information and adjust the health literacy level of the information. Therefore, we shifted our work to identify predictors of the likelihood of older adults starting or stopping a medication and the influence of the source of the information.

## Methods

### Study Design and Sample

We developed a 15-minute web-based experimental survey informed by our previous research, which focused on factors important to older adults when making medication decisions [17-19]. We used Qualtrics panels, which include more than 3 million people, to distribute the survey. We initially pilot-tested the survey by distributing it to 50 older adults. We did not make any changes to the survey. Overall, we aimed to recruit 1200 US adults aged 65 years and older to participate between December 2023 and January 2024. We used quotas for gender (50% female), age (50% aged 65-69 years and 50% aged 70 years and older), race (15% Black, 5% Asian, and 80% White or another race), ethnicity (18% Hispanic), and education (70% less than a bachelor’s degree). We aimed to align our quotas with the prevalence of these groups in the US population. The sampling algorithm continued to invite eligible participants to complete the survey until all quotas were achieved. Survey invitations did not include the study topic to decrease self-selection bias, and strategies such as checking IP addresses were used to prevent individuals from submitting multiple responses.

### Intervention

First, we defined 5 potential sources of information for learning about OTC and prescription medications (Table 1). These included 2 health care professionals (ie, primary care provider [PCP] or pharmacist at a drugstore) and 3 versions of an AI app (ie, connect with electronic health record [EHR] and sends medication recommendations to PCP for approval before sending it to the patient [“EHR-PCP”], connects with EHR and sends a medication recommendation directly to the patient [“EHR-Direct”], or collects information by asking the patient a question and then provides a medication recommendation directly to the patient [“Questions-Direct”]). We developed the descriptions of the AI apps based on semistructured interviews that we had previously conducted with adults to explore their interest in using technology when making OTC medication decisions [11].

**Table 1:** Study design^a^.

Variable	Text description
Source information	There are many ways that you can learn about *over-the-counter and prescription medications*. You may receive a recommendation from a health care professional: You might have an appointment with or message a primary care provider (PCP) who has access to your electronic health record. You might ask a pharmacist at a drugstore who does not have access to your electronic health record. [Page break] Alternatively, you may receive a recommendation from an application (‘app’) that uses *artificial intelligence* (AI) and all of the available *research evidence* to make personalized recommendations for you. One type of app may gather information from your electronic health record such as your health conditions and medications to create a personalized recommendation. The recommendation would be shared with your doctor for approval before your receive the information. [EHR-PCP] A second type of app may gather information from your electronic health record such as your health conditions and medications to create a personalized recommendation. You would receive the recommendation immediately and a copy of the recommendation would be stored in your electronic health record. [EHR-Direct] A third type of app may gather information from you as you answer a series of questions about your health conditions and medications to create a personalized recommendation. You would receive the recommendation immediately. [Questions-Direct]
Medication	Imagine that you are interested in simplifying your medication regimen to avoid unnecessary medications and decrease the risk of side effects. Consider the following three situations and identify how likely you would be to *stop* the medication if recommended by each of the following sources of information. *[randomized order with page breaks]* Imagine that you have taken low-dose aspirin once daily for several years to prevent you from having a heart attack or stroke. You have never had a heart attack or stroke. However, you recently learned that aspirin can increase your risk of bleeding. Imagine that you have taken ranitidine once daily for several years to treat your heartburn symptoms. Your symptoms are well controlled. However, you recently learned that ranitidine contains a cancer-causing chemical. Imagine that you have taken prescription simvastatin once daily for several years to decrease your risk of a heart attack or stroke. Your cholesterol lab values are within the normal range. However, you recently learned that it may not provide as much benefit as you age. You are looking for advice as to whether this information means you should stop taking [medication] How likely would you be to stop the [medication] if recommended by [source of information]? 1 = Not at all likely 2 3 4 5 6 = Extreme likely

^a^Participants received 3 vignettes (ie, aspirin, ranitidine, and simvastatin) in a randomized order. For each vignette, participants indicated how likely they would be to stop the medication if recommended by 5 different sources of information (ie, primary care provider, a pharmacist at a drugstore, “EHR-PCP,” “EHR-Direct,” and “Questions-Direct”).

We presented 3 medication vignettes (ie, aspirin, ranitidine, and simvastatin) to each participant in a randomized order. Aspirin was selected given it is widely accessible as an OTC medication, taken by approximately one-third of older adults without cardiovascular disease, yet not recommended by clinical guidelines given the risks of bleeding [20,21]. Ranitidine was an OTC histamine-2 receptor antagonist, which was withdrawn from the US market in 2020 after being a best-selling medication in the United States, given that it contained a probable carcinogen [22,23]. Simvastatin is a prescription hydroxymethylglutaryl-CoA reductase inhibitor recommended for the primary prevention of cardiovascular events [24,25]. Participants were not able to go backward in the study due to the randomization of the vignettes.

Participants were asked to rate the likelihood that they would stop each medication based on the source of information (6-point Likert scale with “Not at all likely (1)” and “Extremely likely (6)” as scale anchors). We subsequently asked participants to think about the AI app that connects with the EHR and provides immediate, personalized advice about OTC medications with a recommendation stored in the EHR. Participants were asked to rate their level of agreement with statements related to access, trust, nervousness, confusion and discomfort, and preferring to use the app before reaching out to the PCP (5-point Likert scale with “1=Strongly disagree” and “2=Strongly agree (5)” as scale anchors).

### Demographic Characteristics

We collected information about participants’ age, gender, race, ethnicity, and highest level of education. Participants reported their health status using a 5-point Likert scale with response options of “Poor (1),” “Fair (2),” “Good (3),” “Very good (4),” and “Excellent (5),” and their health literacy level was measured by their level of confidence filling out medical forms with response options of “Not at all (1),” “A little bit (2),” “Somewhat (3),” “Quite a bit (4),” and “Excellent (5)” [26-28]. We also asked about their confidence using various forms of technology such as the internet, EHR, and apps (5-point Likert scale with “Not at all confident (1)” and “Very confident (5)” as scale anchors). We included 5 items modified from the technology acceptance model (ie, whether the AI app increases access, trustworthiness, nervousness, confusion and discomfort, and is preferred to contacting a PCP) [29,30].

### Ethical Considerations

The University of Michigan Institutional Review Board deemed this study exempt (HUM00246349). The informed consent information was included on the first page of the survey. All questions were required; however, participants could exit the survey anytime, and all data were collected anonymously. Prospective participants were told the anticipated duration of the survey, where and how long data would be stored, the investigator, and the purpose of the survey. Participants were compensated based on the terms of their panel agreement.

### Statistical Analysis

We calculated descriptive statistics and reported the percentage of participants who reported being extremely likely to stop a medication (score of 6) based on the source of information. High Cronbach α scores indicated that participants responded consistently across medications by the source of information. Therefore, we created an average medication score by the source of information per participant. We used logistic regression to explore demographic characteristics that predict a high likelihood of stopping medications (scores 4-6) compared to those with low scores (scores 1-3). We used linear regression with continuous integer values between “Not at all confident (1)” and “Very confident (5)” to explore demographic characteristics that predict attitudes toward using the Questions-Direct-OTC app. All regression models included the demographic characteristics of age; gender; race; ethnicity; education; health status; health literacy; and confidence using the internet, EHR, and apps. We used a statistical significance level of *P*<.05. All analyses were conducted with R statistical software (version 4.2.2). We reported our study using the Strengthening the Reporting of Observational Studies in Epidemiology (STROBE) checklist and Checklist for Reporting Results of Internet E-Surveys (CHERIES) [31,32].

## Results

The survey was opened by 1318 individuals, of whom 73 participants were excluded due to not meeting the inclusion criteria (eg, aged younger than 65 years) or demographic quotas being filled. The final sample for analysis included 1245 respondents (94% completion rate).

### Demographic Characteristics

Half (n=625, 50%) of the 1245 participants were female, and the median age was 70 (IQR 67-74 years; Table 2). Participants most often reported earning a high school diploma or less (n=391, 32%) or attending trade school, some college, or earning an associate’s degree (n=484, 39%). Participants most frequently reported having good health (n=595, 48%) and being extremely comfortable filling out medical forms. Most participants were confident making phone calls (n=1070, 86%), sending and receiving emails (n=1025, 82%) and SMS text messages (n=992, 80%), and using the internet (n=906, 73%). Fewer participants were confident using an EHR (n=790, 63%), apps (n=769, 62%), social media (n=607, 49%), or wearable devices (n=536, 43%).

**Table 2:** Demographic characteristics of older adult participants (n=1245).

Demographic characteristics			Value
**Age (years), m** **edian (IQR)**			70 (67-74)
**Gender, n (%)**			
	Female	625 (50)	
	Male	619 (50)	
	Transgender	1 (0)	
**Race, n (%)**			
	White	894 (72)	
	Black or African American	195 (16)	
	Asian or Asian American	61 (5)	
	Another race	95 (8)	
**Ethnicity, n (%)**			
	Hispanic	229 (18)	
**Education, n (%)**			
	High school diploma or less	391 (32)	
	Trade school, some college, or associate’s degree	484 (39)	
	Bachelor’s degree	240 (19)	
	Master’s degree or higher	130 (10)	
**Health status, n (%)**			
	Excellent	55 (4)	
	Very good	282 (23)	
	Good	595 (48)	
	Fair	267 (21)	
	Poor	46 (4)	
**Confidence filling out medical forms, n (%)**			
	Extremely	662 (53)	
	Quite a bit	376 (30)	
	Somewhat	142 (11)	
	A little bit	45 (4)	
	Not at all	20 (2)	
**Confidence using technology^a^, n (%)**			
	Phone calls	1070 (86)	
	Emails	1025 (82)	
	SMS text messages	992 (80)	
	Internet	906 (73)	
	Electronic health record	790 (63)	
	Apps	769 (62)	
	Social media	607 (49)	
	Wearable devices	536 (43)	

^a^Participants who selected scores of 4 or 5 on a 5-point Likert scale with scale anchors 1=not at all confident and 5=very confident.

### Likelihood of Stopping Medications

Older adults (n=1245) more frequently reported being extremely likely to stop a medication when the recommendation came from a PCP (range from n=748, 60.1% for aspirin due to a risk of bleeding to n=858, 68.9% for ranitidine due to a cancer-causing chemical; Table 3). In contrast, older adults were less likely to say that they would follow the recommendation from a pharmacist at a drugstore (extremely likely ranging from n=227, 18.2% for simvastatin due to a lack of benefit with age to n=361, 29% for ranitidine).

Older adults were least likely to agree with stopping medication when the recommendation came from an AI app. Between 14.6% (n=182; aspirin) and 23.2% (n=289; ranitidine) of older adults were extremely likely to stop the medication when recommended by the EHR-PCP app. Regardless of integration within the EHR, when the AI app did not include approval by a PCP, only ~10% (n=130, 10.4% for aspirin and n=132, 10.6% simvastatin) and 17% (n=212; ranitidine) of older adults were extremely likely to stop the medication.

In adjusted analyses, older adults who were female as opposed to male were more likely to stop a medication when the recommendation came from a PCP (odds ratio [OR] 1.55, 95% CI 1.18-2.03) or pharmacist (OR 1.27, 95% CI 1.10-1.47; Table 4).

Compared to older adults who were White, older adults who were Black or African American were less likely to stop a medication when the recommendation came from a PCP (OR 0.51, 95% CI 0.37-0.71); however, they were more likely to stop a medication when recommended by the EHR-PCP app (OR 1.42, 95% CI 1.17-1.73), EHR-Direct app (OR 1.38, 95% CI 1.14-1.66), or Questions-Direct app (OR 1.28, 95% CI 1.06-1.54).

Compared to older adults who were White, older adults who identified as another race were less likely to stop a medication when the recommendation came from a pharmacist (OR 0.71, 95% CI 0.57-0.88). However, they were more likely to stop a medication when the recommendation came from the EHR-direct app (OR 1.23, 95% CI 1.00-1.51).

Compared with older adults with less than a bachelor’s degree, older adults with a bachelor’s degree were more likely to stop a medication when the recommendation came from a PCP (OR 1.71, 95% CI 1.23-2.38) or the EHR-PCP app (OR 1.36, 95% CI 1.17-1.58).

There was no difference in the likelihood of stopping a medication by health status when the recommendation came from a PCP. However, higher health status was associated with an increased likelihood of stopping the medication for the other 4 sources, ranging from the EHR-PCP app (OR 1.09, 95% CI 1.01-1.18) to the EHR-Direct app (OR 1.13, 95% CI 1.05-1.23). In contrast, as health literacy increased, older adults were more likely to stop a medication when recommended by a PCP (OR 1.19, 95% CI 1.04-1.36) and less likely to stop a medication when it was recommended by the EHR-PCP app (OR 0.81, 95% CI 0.75-0.88), EHR-Direct app (OR 0.85, 95% CI 0.78-0.92), or Questions-Direct app (OR 0.83, 95% CI 0.77-0.90).

There was no difference in the likelihood of stopping a medication by confidence using the internet except for recommendations by a pharmacist at a drugstore (OR 1.31, 95% CI 1.10-1.56). However, older adults who were more confident using the EHR were more likely to stop a medication when it was recommended by the EHR-PCP app (OR 1.55, 95% CI 1.33-1.80), EHR-Direct app (OR 1.44, 95% CI 1.24-1.67), or Questions-Direct app (OR 1.36, 95% CI 1.16-1.58). Similarly, older adults who were more confident using apps were more likely to stop a medication when it was recommended by the EHR-PCP app (OR 1.49, 95% CI 1.27-1.74), EHR-Direct app (OR 1.38, 95% CI 1.18-1.62), or Questions-Direct app (OR 1.39, 95% CI 1.19-1.64).

**Table 3:** Older adult’s likelihood of stopping a medication based on the source of the recommendation and score (n=1245)^a^.

Source of information					Low-dose aspirin (risk of bleeding), n (%)	Ranitidine (cancer-causing chemical), n (%)	Simvastatin (lack of benefit with age), n (%)
**PCP^b^**							
	1=Not at all likely				42 (3.4)	21 (1.7)	20 (1.6)
	2				22 (1.8)	8 (0.6)	15 (1.2)
	3				51 (4.1)	35 (2.8)	43 (3.5)
	4				104 (8.4)	78 (6.3)	100 (8)
	5				278 (22.3)	245 (19.7)	279 (22.4)
	6=Extremely likely				748 (60.1)	858 (68.9)	788 (63.3)
**Pharmacist at a drugstore**							
		1=Not at all likely			137 (11)	104 (8.4)	122 (9.8)
		2			105 (8.4)	68 (5.5)	105 (8.4)
		3			190 (15.3)	156 (12.5)	218 (17.5)
		4			294 (23.6)	261 (21)	292 (23.5)
		5			286 (23)	295 (23.7)	281 (22.6)
		6=Extremely likely			233 (18.7)	361 (29)	227 (18.2)
**AI^c^ app that connects with EHR^d^ and provides personalized advice to PCP for approval prior to sharing with you**							
				1=Not at all likely	270 (21.7)	230 (18.5)	266 (21.4)
				2	122 (9.8)	95 (7.6)	117 (9.4)
				3	200 (16.1)	178 (14.3)	193 (15.5)
				4	237 (19)	214 (17.2)	252 (20.2)
				5	234 (18.8)	239 (19.2)	217 (17.4)
				6=Extremely likely	182 (14.6)	289 (23.2)	200 (16.1)
**AI app with EHR access that provides personalized advice directly to you**							
			1=Not at all likely		323 (25.9)	270 (21.7)	310 (24.9)
			2		151 (12.1)	135 (10.8)	175 (14.1)
			3		261 (21)	218 (17.5)	242 (19.4)
			4		231 (18.6)	237 (19)	243 (19.5)
			5		149 (12)	173 (13.9)	157 (12.6)
			6=Extremely likely		130 (10.4)	212 (17)	118 (9.5)
**AI app that collects information by asking you questions and provides personalized advice directly to you**							
		1=Not at all likely			337 (27.1)	281 (22.6)	318 (25.5)
		2			169 (13.6)	151 (21.1)	165 (13.3)
		3			248 (19.9)	222 (17.8)	271 (21.8)
		4			228 (18.3)	208 (16.7)	240 (19.3)
		5			142 (11.4)	179 (14.4)	119 (9.6)
		6=Extremely likely			121 (9.7)	204 (16.4)	132 (10.6)

^a^This table shows the percentage of participants who selected each score on a 6-point Likert scale with 1=not at all likely and 6=extremely likely to stop the medication as scale anchors.

^b^PCP: primary care provider.

^c^AI: artificial intelligence.

^d^EHR: electronic health record.

**Table 4:** Demographic predictors of being likely to stop a medication based on the source of the recommendation using logistic regression (n=1245)^a^.

				PCP^b^		Pharmacist at a drugstore		AI^c^ app with EHR^d^ access and PCP approval^e^		AI app with EHR access and direct recommendation^f^		AI app without EHR access and direct recommendation^g^
**Age, OR^h^ (95% CI)**				1.03 (1.00-1.06)		0.99 (0.98-1.00)		1.00 (0.99-1.01)		1.01 (1.00-1.03)		1.01 (1.00-1.02)
**Gender, OR (95% CI)**												
		Male	REF^i^		REF		REF		REF		REF	
		Female	1.55 (1.18-2.03)		1.27 (1.10-1.47)		1.14 (0.99-1.31)		1.01 (0.88-1.16)		1.04 (0.91-1.20)	
**Race, OR (95% CI)**												
		White	REF		REF		REF		REF		REF	
		Black of African American	0.51 (0.37-0.71)		0.90 (0.74-1.10)		1.42 (1.17-1.73)		1.38 (1.14-1.66)		1.28 (1.06-1.54)	
		Another race	0.93 (0.60-1.41)		0.71 (0.57-0.88)		1.21 (0.98-1.49)		1.23 (1.00-1.51)		1.17 (0.95-1.44)	
**Ethnicity, OR (95% CI)**												
		Non-Hispanic	REF		REF		REF		REF		REF	
		Hispanic	0.88 (0.61-1.27)		1.15 (0.95-1.40)		0.98 (0.82-1.17)		0.96 (0.81-1.15)		1.04 (0.87-1.24)	
**Education, OR (95% CI)**												
		<Bachelor’s degree	REF		REF		REF		REF		REF	
		≥Bachelor’s degree	1.71 (1.23-2.38)		0.97 (0.83-1.14)		1.36 (1.17-1.58)		1.14 (0.98-1.32)		1.05 (0.91-1.22)	
		Health status	1.03 (0.89-1.21)		1.13 (1.04-1.22)		1.09 (1.01-1.18)		1.13 (1.05-1.23)		1.13 (1.05-1.22)	
		Health literacy	1.19 (1.04-1.36)		1.02 (0.94-1.11)		0.81 (0.75-0.88)		0.85 (0.78-0.92)		0.83 (0.77-0.90)	
**Confidence using internet^j^, OR (95% CI)**												
		Low	REF		REF		REF		REF		REF	
		High	1.36 (1.00-1.85)		1.31 (1.10-1.56)		1.16 (0.98-1.37)		1.06 (0.90-1.26)		1.13 (0.96-1.34)	
**Confidence using electronic health record^j^, OR (95% CI)**												
		Low	REF		REF		REF		REF		REF	
		High	1.22 (0.92-1.63)		1.13 (0.96-1.32)		1.55 (1.33-1.80)		1.44 (1.24-1.67)		1.36 (1.16-1.58)	
**Confidence using apps^j^, OR (95% CI)**												
	Low		REF		REF		REF		REF		REF	
	High		1.30 (0.95-1.78)		1.13 (0.96-1.32)		1.49 (1.27-1.74)		1.38 (1.18-1.62)		1.39 (1.19-1.64)	

^a^6-point Likert scale: 1=not at all likely, 6=extremely likely; participants with scores 4-6 were classified as “likely.”

^b^PCP: primary care provider.

^c^AI: artificial intelligence.

^d^EHR: electronic health record.

^e^AI app that connects with your EHR and provides personalized advice to your PCP for approval before sharing the recommendation with you.

^f^AI app that connects with your EHR and provides personalized advice directly to you.

^g^AI app that collects information by asking you questions and provides personalized advice directly to you.

^h^OR: odds ratio.

^i^REF: reference category.

^j^5-Point Likert scale: 1=not at all confident, 5=very confident; participants were categorized as no (scores 1-3) or yes (scores 4-5).

### Attitudes Toward Using an AI App

We asked participants about their attitudes toward using an AI app with EHR access to provide immediate, personalized advice about OTC medications (Table 5). Compared to participants who were White, participants who were Black or African American were more likely to report that this technology would improve access to information (=0.32, 95% CI 0.13-0.51), they would trust the personalized advice (=0.24, 95% CI 0.06-0.43), and it would not lead to confusion or discomfort (=0.21, 95% CI 0.00-0.42) as it relates to advice about OTC medications. Compared to White participants, participants of another race were likelier to report that the app would not lead to confusion or discomfort (=0.27, 95% CI 0.04-0.49). There were no differences by age, gender, or ethnicity in attitudes toward using an AI app.

Compared to participants with less than a bachelor’s degree, participants with a bachelor’s degree or higher were more likely to report that the app would improve access to medication information (=0.21, 95% CI 0.06-0.35), they would trust it (=0.20, 95% CI 0.06-0.35), and it would not lead to confusion or discomfort (=0.20, 95% CI 0.04-0.37). In contrast, participants who had higher health literacy, as evidenced by higher confidence in filling out medical forms, were less likely to believe that the app would improve access (=–0.10, 95% CI –0.18 to –0.02), they were less likely to trust the advice (=–0.14, 95% CI –0.22 to –0.07), and they were less likely to report that they would prefer to use the app before reaching out to their PCP (=–0.17, 95% CI –0.24 to –0.09). There were no differences by self-reported health status.

Compared to participants who reported low confidence, participants who reported high confidence using the internet were likelier to report that an app would increase their access to information about OTC medications (=0.22, 95% CI 0.06-0.39). Participants who reported high confidence using an EHR reported more positive perceptions of the app across all domains, while those who reported high confidence using apps reported more positive perceptions across all domains except nervousness, which showed negative perceptions.

**Table 5:** Demographic predictors of attitudes towards the use of an app that uses artificial intelligence (AI) along with the electronic health record to provide immediate, personalized advice about over-the-counter medications with a recommendation stored in the electronic health record using linear regression (n=1245).

			Access: using an AI app that provides personalized advice would improve my access to information about medications that are right for me		Trust: I would trust an AI app that provides personalized advice about medications that are right for me		(Not) nervous: using an AI app that provides personalized advice would make me feel (not) nervous^a^		(Not) uncomfortable: using an AI app that provides personalized advice would (not) make me confused and uncomfortable^a^		Prefer: whenever I need advice about medications, I would prefer to use an AI app that provides personalized advice before reaching out to my primary care provider with the same question
**Age, OR^b^ (95% CI)**			0.00 (–0.01 to 0.01)		0.00 (–0.01 to 0.01)		–0.01 (–0.03 to 0.00)		–0.01 (–0.02 to 0.00)		–0.01 (–0.02 to 0.01)
**Gender, OR (95% CI)**											
	Male	REF^c^		REF		REF		REF		REF	
	Female	–0.04 (–0.18 to 0.09)		–0.07 (–0.20 to 0.06)		–0.10 (–0.26 to 0.05)		–0.19 (–0.34 to –0.03)		–0.08 (–0.22 to 0.05)	
**Race, OR (95% CI)**											
	White or Caucasian	REF		REF		REF		REF		REF	
	Black of African American	0.32 (0.13 to 0.51)		0.24 (0.06 to 0.43)		0.20 (–0.01 to 0.40)		0.21 (0.00 to 0.42)		0.10 (–0.09 to 0.28)	
	Another race	0.19 (–0.02 to 0.39)		0.05 (–0.15 to 0.25)		0.03 (–0.20 to 0.26)		0.27 (0.04 to 0.49)		0.16 (–0.04 to 0.37)	
**Ethnicity, OR (95% CI)**											
	Non-Hispanic	REF		REF		REF		REF		REF	
	Hispanic	–0.09 (–0.27 to 0.09)		0.08 (–0.09 to 0.25)		0.10 (–0.09 to 0.30)		0.19 (–0.01 to 0.38)		–0.14 (–0.31 to 0.04)	
**Education, OR (95% CI)**											
	<Bachelor’s degree	REF		REF		REF		REF		REF	
	≥Bachelor’s degree	0.21 (0.06 to 0.35)		0.20 (0.06 to 0.35)		0.13 (–0.04 to 0.29)		0.20 (0.04 to 0.37)		0.10 (–0.04 to 0.25)	
	Health status	–0.02 (–0.10 to 0.06)		0.04 (–0.04 to 0.11)		0.02 (–0.07 to 0.10)		0.04 (–0.05 to 0.13)		0.01 (–0.06 to 0.09)	
	Health literacy	–0.10 (–0.18 to –0.02)		–0.14 (–0.22 to –0.07)		0.03 (–0.05 to 0.12)		0.04 (–0.04 to 0.13)		–0.17 (–0.24 to –0.09)	
**Confidence using internet^d^, OR (95% CI)**											
	Low	REF		REF		REF		REF		REF	
	High	0.22 (0.06 to 0.39)		0.16 (0.00 to 0.32)		0.14 (–0.04 to 0.32)		0.01 (–0.18 to 0.19)		0.05 (–0.11 to 0.22)	
**Confidence using electronic health record^d^, OR (95% CI)**											
	Low	REF		REF		REF		REF		REF	
	High	0.25 (0.10 to 0.40)		0.21 (0.07 to 0.36)		0.23 (0.07 to 0.40)		0.32 (0.15 to 0.48)		0.18 (0.04 to 0.33)	
**Confidence using apps^d^, OR (95% CI)**											
	Low	REF		REF		REF		REF		REF	
	High	0.53 (0.37 to 0.69)		0.47 (0.31 to 0.62)		0.17 (0.00 to 0.35)		0.35 (0.18 to 0.53)		0.38 (0.22 to 0.53)	

^a^Ratings for “nervous” and “uncomfortable” were reverse-coded for analysis so that higher values represent positive outcomes in all regressions.

^c^OR: odds ratio.

^c^REF: reference category.

^d^5-Point Likert scale: 1=not at all confident, 5=very confident; participants were categorized as low (scores 1-3) or high (scores 4-5).

## Discussion

### Principal Findings

Older adults are increasingly using technology, such as smartphone apps and virtual medical appointments, as part of managing their health [9]. In our study, we asked older adults to imagine interacting with an AI app that provides personalized medication recommendations. In this study using hypothetical vignettes, most older adults preferred to follow a recommendation to stop a medication when provided by a PCP rather than a pharmacist at a drugstore or via an AI app. However, older adults who were Black or African American reported a lower likelihood of following a recommendation from a PCP and a higher likelihood of following a medication recommendation from an AI app. AI-based tools are quickly being integrated into health care systems to diagnose diseases, suggest evidence-based treatment plans, and help health care professionals make medical decisions [33-36]. However, older adults have expressed reservations about their use as part of the medical decision-making process [37]. To our knowledge, this is the first quantitative study to explore older adults’ attitudes about using a patient-facing AI-app to help make medication decisions.

### Comparison With Prior Work

Previous studies identified that older adults frequently report being willing to stop a medication if their doctor said it was possible [38,39]. Tools such as the Anticholinergic Burden Calculator, Beers Criteria, and MedStopper provide evidence-based recommendations to clinicians about potentially inappropriate medications [40-42]. Furthermore, clinical decision support systems are increasingly used within health systems to assist clinicians in making medication decisions [43-45]. Rao et al [46] recently tested whether a publicly available LLM could provide appropriate medication discontinuation recommendations and concluded that specially trained LLMs may be useful for clinicians.

Doctors are among the most trusted professions in the United States [47]. Older adults expect their doctors to provide appropriate medication recommendations and trust that information when they make decisions [5,48]. Increased patient trust is associated with improved satisfaction with treatment, health outcomes, and quality of life [49]. Unfortunately, engaging in shared decision-making about potentially stopping medication in routine clinical practice is difficult due to limited time, competing priorities, and the increasing lack of continuity of care, leading to a breakdown in relationships between patients and health care professionals [50-52]. While adults in the United States have expressed concerns about clinicians relying on AI when making health care decisions, we sought to explore older adults’ perceptions of using AI tools specifically to support medication discontinuation recommendations [53]. Across the 3 AI app hypothetical vignettes that we presented, in 47% of vignettes, older adults agreed (scores 4-6) with a recommendation to stop the medication, of which 14% of ratings were extremely likely (score 6). This indicates that some older adults may be willing to use this technology but have reservations about automatically following its recommendations. This caution is warranted, given the lack of regulations on the use of AI in health care [54].

We observed that older adults’ likelihood of stopping a medication varied by source of information; however, there was little variation across different therapeutic classes and rationales for discontinuing a medication. This aligns with a previous web-based survey of older adults using hypothetical vignettes that found that characteristics of older adults, as opposed to medication-specific characteristics, predicted willingness to stop a medication [55]. In contrast, a larger survey of older adults in 4 countries using hypothetical vignettes identified that the medication type and rationale for discontinuation were important factors in the decision-making process [17]. Additional research is needed to assess older adults’ willingness to accept recommendations provided by an AI app with a broader range of prescription and OTC medications and rationales for discontinuation.

We were surprised that individuals with low health literacy were more likely to accept a recommendation for a medication when it was provided by an AI tool. It is possible that individuals with low health literacy were more interested in using technology, given the stigma they may experience when interacting with the traditional health care system [56,57]. However, older adults with lower health literacy may need additional support to use this type of web-based tool effectively. We observed that older adults with high confidence in using the EHR and apps were more likely to have positive perceptions of using an AI app to make medication decisions and to follow its recommendation to stop a medication. This is an important area for continuing research, given the potential for increased inequalities due to inadequate digital health literacy [58,59].

In our study, older adults who were Black or African American (as compared to White) were less likely to agree with stopping a medication when a PCP recommended it. This aligns with literature that reports that Black or African American adults are less likely to trust their doctor [60,61]. This may be due to the negative experiences and discrimination that Black or African American adults report continuing to occur in health care settings [62]. Health care providers should engage more frequently in shared decision-making conversations with all patients, especially those from minoritized communities, taking into consideration cultural values, preferences, and what matters most to them, as these may be important factors that play a role in the decision-making process for initiating or discontinuing prescribed treatment strategies. In contrast, we found that older adults who were Black or African American were more likely to agree with stopping a medication when an AI app recommended it. While Black or African American individuals are less likely to have internet access, those who do have access use social media, which is often available via apps, at higher rates than other racial groups [63-65]. AI apps may have the potential to improve accessibility to personalized health care information, but they also include biases and stereotypes that could amplify existing health care disparities [66].

### Limitations

The primary limitation of our study is that we asked participants to share their perceptions about hypothetical AI apps with limited written descriptions. Further research is needed to determine whether older adults who reported having an interest in using an AI app would use the tool when making real-world medication decisions. Second, we acknowledge that our study was conducted as a web-based survey, which may have resulted in participation from older adults who were relatively comfortable with technology or who responded positively to questions about technology due to social desirability bias. Third, our findings may not be generalizable to all medications, given that agreement with stopping a medication is influenced by factors such as the type of medication, rationale for recommending that the medication be discontinued, and contextual factors related to the individual patient [17-19,67-70]. Finally, this study was limited to adults aged 65 years and older; additional research is needed to explore to what extent younger adults prefer to use AI apps when making medication decisions.

### Conclusions

It is important to understand older adults’ perceptions of AI tools for health care to inform the development of user-friendly, reliable apps that will be useful in the real-world setting. Our study findings suggest that older adults have reservations about stopping a medication when an AI app recommends it. However, individuals who are Black or African American, have higher self-reported health, or have higher confidence in using an EHR or apps may be receptive to AI-based medication recommendations. Future research is needed to explore whether patient-facing, AI-based clinical decision support systems may complement the traditional health care system and increase access to accurate, personalized medication advice to improve clinical and patient-centered outcomes.

## Abbreviations

AI: artificial intelligence
CHERIES: Checklist for Reporting Results of Internet E-Surveys
EHR: electronic health record
EHR-Direct: artificial intelligence app that connects with the electronic health record and directly provides advice to the consumer
EHR-PCP: artificial intelligence app that connects with the electronic health record and provides advice to the primary care provider for approval before sharing the recommendation with the consumer
LLM: large language mode
OR: odds ratio
OTC: over-the-counter
PCP: primary care provider
Questions-Direct: artificial intelligence app that asks questions and provides advice directly to the consumer
STROBE: Strengthening the Reporting of Observational Studies in Epidemiology

## Data Availability

The datasets generated and analyzed during this study are available from the corresponding author upon reasonable request.
